# Uncovering the Effect of Lattice Strain and Oxygen Deficiency on Electrocatalytic Activity of Perovskite Cobaltite Thin Films

**DOI:** 10.1002/advs.201801898

**Published:** 2019-01-30

**Authors:** Xi Liu, Lei Zhang, Yun Zheng, Zheng Guo, Yunmin Zhu, Huijun Chen, Fei Li, Peipei Liu, Bo Yu, Xinwei Wang, Jiang Liu, Yan Chen, Meilin Liu

**Affiliations:** ^1^ Guangzhou Key Laboratory for Surface Chemistry of Energy Materials Guangdong Engineering and Technology and Research Center for Surface Chemistry of Energy Materials State Key Laboratory of Pulp and Paper Engineering School of Environment and Energy South China University of Technology Guangzhou Guangdong 510006 China; ^2^ Materials Science and Engineering Georgia Institute of Technology Atlanta GA USA; ^3^ Institute of Nuclear and New Energy Technology Tsinghua University Beijing China; ^4^ School of Advanced Materials Shenzhen Graduate School Peking University Shenzhen China

**Keywords:** oxygen defects, perovskite oxides, strain, surface reaction, thin films

## Abstract

Developing cost effective electrocatalysts with high oxygen evolution reaction (OER) activity is essential for large‐scale application of many electrochemical energy systems. Although the impacts of either lattice strain or oxygen defects on the OER performance of oxide catalysts have been extensively investigated, the effects of both factors are normally treated separately. In this work, the coupled effects of both strain and oxygen deficiency on the electrocatalytic activity of La_0.7_Sr_0.3_CoO_3−δ_ (LSC) thin films grown on single crystal substrates (LaAlO3 (LAO) and SrTiO3 (STO)) are investigated. Electrochemical tests show that the OER activities of LSC films are higher under compression than under tension, and are diminished as oxygen vacancies are introduced by vacuum annealing. Both experimental and computational results indicate that the LSC films under tension (e.g., LSC/STO) have larger oxygen deficiency than the films under compression (e.g., LSC/LAO), which attribute to smaller oxygen vacancy formation energy. Such strain‐induced excessive oxygen vacancies in the LSC/STO increases the *e_g_* state occupancy and enlarges the energy gap between the O 2p and Co 3d band, resulting in lower OER activity. Understanding the critical role of strain–defect coupling is important for achieving the rational design of highly active and durable catalysts for energy devices.

## 1 Introduction

The sluggish kinetics of the oxygen evolution reaction (OER) severely limits the performance of many electrochemical energy conversion and storage systems, such as metal air batteries and water electrolysis.^1^ Cost‐effective transition metal oxides have been widely investigated as promising OER catalysts to replace noble metal–based materials such as IrO_2_,^2^ RuO_2_,^3^ Pt,^4^ Pd,^5^ and Au.^6^ Great effort has been devoted to improving the activity and stability of oxide catalysts. Lattice strain has recently attracted much attention as a tuning knob to improve the performance of oxide catalysts.^7^ For instance, Stoerzinger et al. demonstrated that moderate tensile strain enhanced the oxygen electrocatalytic activity of LaCoO_3_.[[qv: 7a]] A compressively strained LaNiO_3_[[qv: 7b]] and LaCoO_3_[[qv: 7c]] were reported to show enhanced OER activity due to strain‐induced changes in the electronic structure. Besides tuning the lattice strain, manipulating anionic defects present another approach that can be used to enhance the activity of electrocatalysts.^8^ Xu et al. reported significantly improved OER activity of Co_3_O_4_ with abundant oxygen vacancies created by plasma treatment.[[qv: 8a]] Du et al. found that moderate oxygen‐defective CaMnO_3−δ_ with oxygen nonstoichiometry (δ) of 0.25 showed the highest OER activity.[[qv: 8b]] While Lyu et al. showed that CaMn_0.75_Nb_0.25_O_3−δ_ with excess oxygen vacancies exhibited better OER activity,[[qv: 8c]] Wu et al. observed degraded OER performance of La_0.6_Ca_0.4_CoO_3−δ_ after introducing oxygen vacancies by heat treatment.[[qv: 8d]]

Although changing redox processes by adjusting the strain states^7^ and the oxygen defects^8^ in oxide catalysts have been extensively investigated, the impacts of the strain and the oxygen defects are normally treated independently in these studies. Recently researches demonstrated that the lattice strain and the oxygen defect chemistry are strongly coupled with each other.^9^ For instance, the stability and dynamics of oxygen anions in SrCoO_3_ thin films are found to depend sensitively on the epitaxial strain.[[qv: 9b]] A large amount of oxygen vacancies were observed in CeO_2_ thin films under a large tensile strain.[[qv: 9c]] Strain introduced changes in oxygen defect chemistry were found to strongly influence the hydrogen oxidation reaction activity of Nd_2_NiO_4_ at elevated temperature.[[qv: 9f]] Therefore, to rationally design high performance oxide catalysts, it is essential to understand the coupling effect between the lattice strain and oxygen defects, and their combined impact on the electrocatalytic activity.

In this work, we investigated the coupled effects of lattice strain and oxygen defect chemistry on surface activity of cobaltite oxide thin films. La_0.7_Sr_0.3_CoO_3−δ_ (LSC) thin films grown by pulsed laser deposition (PLD) on single crystal substrates LaAlO_3_ (LAO) (100) and SrTiO_3_ (STO) (100) with different lattice parameters were used as the model systems. As confirmed by high resolution X‐ray diffraction (HRXRD), the LSC deposited on STO (LSC/STO) and LAO (LSC/LAO) presented in‐plane tensile strain and in‐plane compressive strain, respectively. Electrochemical test showed that both the strain and the oxygen defects critically impact on the OER activity of LSC thin films. The LSC/LAO with compressive strain showed higher OER activity than the LSC/STO with tensile strain. Excessive oxygen vacancies introduced by vacuum annealing were found to degrade the OER activity of both LSC/STO and LSC/LAO. HRXRD and X‐ray photoelectron spectroscopy (XPS) results suggested the LSC/STO to have more oxygen vacancies than the LSC/LAO, due largely to smaller oxygen vacancy formation energy for LSC/STO predicted by first‐principles calculations. These excessive oxygen vacancies in LSC/STO resulted in higher *e_g_* states occupancy and larger energy gap of O 2p and Co 3d band center (Δ), resulting in lower OER activity for LSC/STO than LSC/LAO. Our results showed that it is critical to consider strain–defect coupling effects when lattice strain and oxygen nonstoichiometry were used as design parameters to synthesize high performance electrocatalysts.

## 2 Results and Discussion

### 2.1 Effect of Strain on the OER Activity of LSC Films

**Figure 1:**
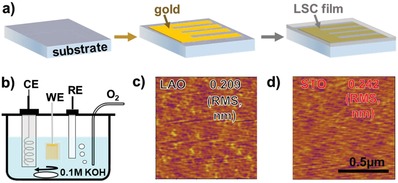
a) Illustration of sample preparation process for LSC thin film electrode. A gold pattern was embedded into the thin film to serve as the current collector for electrochemical measurement; b) illustration of the OER measurement sets up with the film as the WE. c,d) AFM images of LSC model thin films grown on LAO (c) and STO (b) substrates. The surface roughness is less than 1 nm.

Due to the lattice mismatch, oxide thin films that are epitaxially grown on substrates with different lattice parameters present very different strain states. In this work, STO and LAO single crystal substrates with lattice parameters of 3.905 and 3.793 Å[[qv: 9d]] were used to tune the lattice strain of LSC thin films. A pseudo‐cubic lattice constant of *a*_pc_ (*b*_pc_) = 3.849 Å for LSC bulk crystal^10^ led to in‐plane compressive and tensile strain in LSC films epitaxially grown on LAO and STO substrates, respectively. A gold pattern was deposited on the substrate by sputtering before the films (**Figure** **1**a), which were served as current collectors for electrochemical measurement (Figure 1b). As confirmed by HRXRD results shown later, the model LSC thin films could still sustain desired in‐plane strains with Au current collector embedded. All the characterization results shown in this manuscript were carried out on the samples with embedded gold current collector. Using atomic force microscopy (AFM), the surface of LSC films grown on both STO and LAO were found to be very smooth with roughness less than 1 nm (Figure 1c,d).

**Figure 2:**
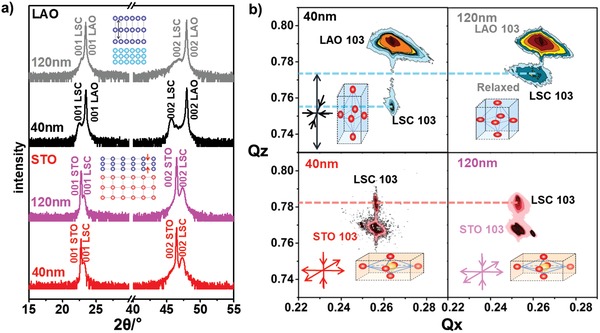
a) HRXRD 2θ–ω scans of LSC/STO and LSC/LAO with two different thicknesses (40 and 120 nm): STO (40 nm, red), STO (120 nm, pink), LAO (120 nm, gray), and LAO (40 nm, black). The inset illustrations show the crystal structure of the thin and substrates. The out‐of‐plane tensile and compressive strain for LSC/LAO and LSC/STO are marked by the blue and red arrows. b) HRXRD RSM results for of LSC/LAO (red) and LSC/STO (blue) with two different thicknesses (40 and 120 nm). The dashed line indicated that the average out‐of‐plane lattice. The black arrows in the inset figures show the in‐plane compressive and out‐of‐plane tensile strain for LSC/LAO; the red arrows in the inset figures show the in‐plane tensile and out‐of‐plane compressive strain for LSC/STO.

The crystal structure and the strain states of the LSC films were characterized by HRXRD 2θ–ω scans and reciprocal‐space mapping (RSM). The 2θ–ω scans (**Figure** **2**a) of LSC/STO and LSC/LAO with two different thicknesses (40 and 120 nm) exhibited only 00*l* peaks, indicating that LSC films were with (00l) orientation regardless of substrate and thickness. Due to the in‐plane strain introduced by the substrates, the LSC/STO exhibited smaller out‐of‐plane lattice parameter than the LSC/LAO did, leading to a higher 2θ value for the 001 and the 002 peaks of LSC/STO in the 2θ–ω scans (Figure 2a). To determine the in‐plane lattice parameter of the LSC thin films, RSM measurements were carried out around the 103 Bragg reflections of the LSC thin films (Figure 2b). For the 40 nm LSC thin films, the in‐plane lattice parameter (*Q_x_*) matched that of the substrates, indicating that the films were fully strained by the substrate. As the film thickness increased to 120 nm, the strain in the LSC film was found to be partially relaxed, leading to a different in‐plane lattice parameter of the film from the substrate (Figure 2b). Such relaxation is more apparently for LSC/LAO with in‐plane tensile strain. This result is consistent with previous reports, which showed that the thin films with in‐plane compressive strain were more likely to form defects such as dislocations to release the lattice strain compared with the ones with in‐plane tensile strain.^11^ The RSM results showed that the 40 nm LSC/LAO was with in‐plane compressive strain of −1.45% and out‐of‐plane tensile strain of 3.27%, while LSC/STO was with in‐plane tensile strain of 1.45% and out‐of‐plane compressive strain of −0.44%. For 120 nm films, LSC/LAO held −0.63% in‐plane strain and 1.11% out‐of‐plane strain, while LSC/STO held 1.41% in‐plane strain and −0.38% out‐of‐plane strain. The STO and LAO substrates showed negligible contribution to the OER activity of the LSC thin film samples (Figure S1, Supporting Information).

**Figure 3:**
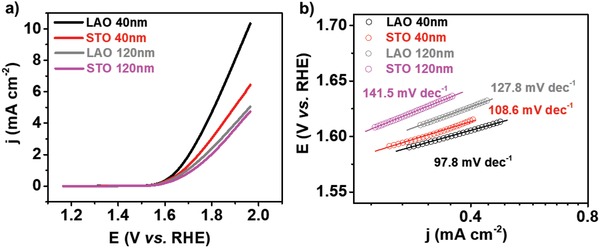
a) LSV curves and b) Tafel plots for the OER on 40 and 120 nm LSC films grown on STO and LAO substrates.

The OER activity of the LSC thin films with different strain states were evaluated using linear sweep voltammetry (LSV) in the OER region (from 1.2 to 2.0 V vs Reversible Hydrogen Electrode (RHE)). As shown in **Figure** **3**a, the 40 nm LSC/LAO showed considerably higher current density and lower onset potential than the LSC/STO. Consistently, the Tafel slope (Figure 3b) for the 40 nm LSC/LAO (97.8 mV dec^−1^) was smaller than that LSC/STO (108.6 mV dec^−1^). These results showed that the 40 nm LSC/LAO with a −1.45% in‐plane compressive strain exhibited much better OER activity than that LSC/STO with a 1.45% in‐plane tensile strain. The 120 nm LSC showed smaller current density and larger Tafel slope than that of 40 nm ones (Figure 3), while the resistances of the liquid electrolyte obtained by electrochemical impedance spectroscopy were similar for the films with different thickness (Figure S3, Supporting Information). The worse performance of the LSC with larger thickness (Figure 2a,b) is likely due to the increased charge transfer resistance during OER process as previously reported by Stoerzinger et al.[[qv: 7a]] In accord with results obtained for the 40 nm LSC thin films, the 120 nm LSC/LAO (−0.63% in‐plane compressive strain) also presented better OER activity than 120 nm LSC/STO (1.41% in‐plane tensile strain), with higher current density (Figure 3a) and smaller Tafel slope (Figure 3b). All the electrochemical measurement results shown above consistently indicated that the LSC/LAO with compressive strain exhibited more enhanced OER activity compared with the LSC/STO with tensile strain. Considering that the strain introduced by the substrate can be better sustained in the thinner films, all the discussion in the following part of this manuscript is focused on the films with 40 nm thickness.

### 2.2 Impact of Oxygen Defects on the OER Activity of LSC Thin Films

Besides the influence of the strain states, the impact of oxygen defects on the OER activity of LSC thin films were also quantified by electrochemical measurement. Oxygen vacancies were introduced into the 40 nm LSC/LAO and LSC/STO thin films by annealing the films in an ultrahigh vacuum chamber at 300 °C for 2 h under a base pressure of 10^−9^ mbar. Because of the small volumes, the commonly used approaches for characterizing oxygen nonstoichiometry in bulk materials, such as thermogravimetric analysis and dilatometry, cannot be applied to thin films. In this work, we carried out HRXRD and XPS to probe the lattice parameters and the valance states of cobalt, and the obtained information were used to indirectly determine the amount of oxygen vacancies in the LSC thin films.^12^

**Figure 4:**
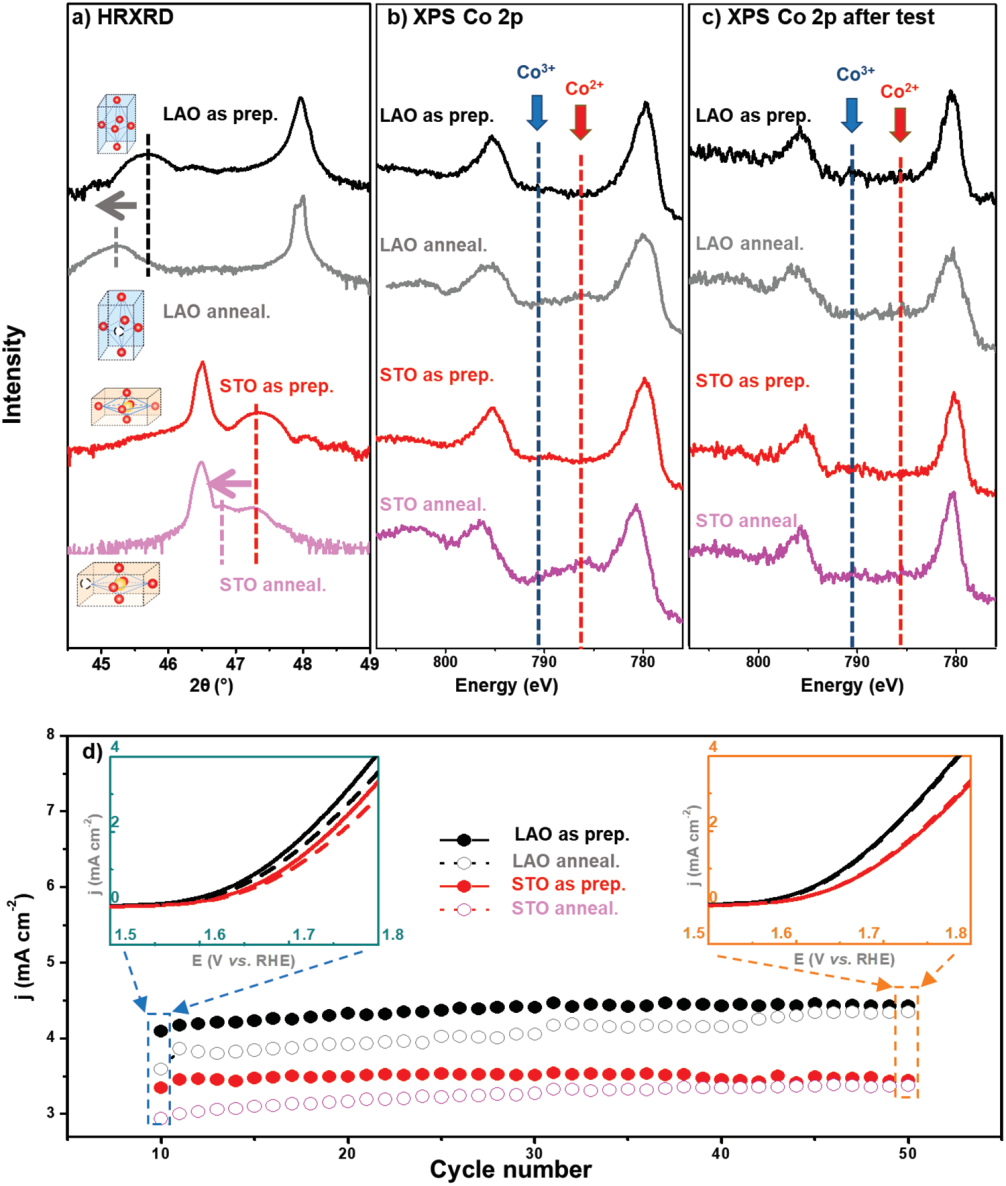
a) HRXRD 2θ–ω scans of LSC films in their as‐prepared states and after vacuum annealing. The arrow showed the shift of the film peaks after vacuum annealing, indicating the chemical expansion introduced by oxygen vacancy formation. b) Co 2p XPS spectra for as‐prepared and vacuum annealed LSC films before (b) and (c) after long time OER test. The dash line marked the Co 2p 3/2 satellite peaks position for Co^2+^ and Co^3+^; d) scatter plots of the current density at 1.8 V versus RHE obtained from each LSV test for LSC/STO and LSC/LAO in their as‐prepared state (solid dot) and after vacuum annealing (hollow dot). Inset green and red figures were LSV curves for as‐prepared and vacuum annealed LSC films obtained in the 10th cycles (left, green) and the 50th cycle (right, orange), respectively.

The HRXRD results showed that the LSC films subjected to vacuum annealing remained in 001 orientation without any secondary phase formation. As shown in **Figure** **4**a, the film peaks shifted to lower 2θ values (Figure 4a), suggesting an expansion in the LSC lattice after vacuum annealing. This effect is associated with the chemical expansions that were widely observed in metal oxides.^12^, ^13^ **Table** **1** lists the out‐of‐plane lattice parameter (*c*) and the unit cell volume (*V*) for the LSC/STO and the LSC/LAO before and after vacuum annealing. The increase of *c* parameter and *V* after annealing indicates the formation of oxygen vacancies in LSC films.[[qv: 12,13b,c]] The unit cell volume of the LSC/LAO was found to be smaller than that for LSC/STO for both as‐prepared states and after annealing, suggesting a potentially less oxygen vacancies in the LSC/LAO than the LSC/STO. To obtain a more quantitative comparison of the oxygen vacancy densities in the LSC/STO and the LSC/LAO, we calculated the relative difference in oxygen nonstoichiometric of all the film (Δδ) with respect to the as‐prepared LSC/LAO (the film with the smallest unit cell volume), by adapting the oxygen vacancy chemical expansivity (*β_C_*) value quantified by Chen et al. for LSC family of materials.[[qv: 13b]] More details about the calculation can be found in Section S1 in the Supporting Information. As shown in Table 1, LSC/STO with tensile strain showed more oxygen vacancy than that in LSC/LAO. The reason for such difference is the smaller oxygen vacancy formation energy of LSC/STO with tensile strain, which will be discussed in more detail in the latter session.

**Table 1:** Comparison of out‐of‐plane lattice parameter, unit cell volume, changes in unit cell volume, and oxygen nonstoichiometry with respect to as‐prepared LSC/LAO film. The oxygen nonstoichiometry was calculated using the chemical expansion efficiency quantified by Chen et al. for LSC family of materials[[qv: 13b]]

Samples	Out‐of‐plane lattice parameter *c* [Å]	*V* [Å^3^]	Δ*V V* ^−1^	Δδ
LAO as prep	3.97	56.86	–	–
LAO anneal.	4.01	57.41	0.010	0.131
STO as prep	3.84	58.52	0.029	0.318
STO anneal.	3.88	59.14	0.040	0.401

The Co valence states of LSC thin film before and after vacuum annealing were characterized by XPS measurement. The XPS Co 2p spectra contained two main peaks, Co 2p^1/2^ and Co 2p^3/2^, corresponding to the spin‐orbital couplings, and two satellite peaks located at higher binding energy of the main peak, corresponding to the electrons transferred from the O 2p to the Co 3d orbital, which screen the electric field of the holes in the Co 2p orbital created during the photoelectron emission process. The shape and position of the satellite peaks, particularly the Co 2p^3/2^ satellite peak, are widely used to identify the valence state of Co.^12^, ^14^ Under the preparation conditions of the LSC thin films, the valence states of Co were reported to be dominantly 3+ with a small amount of 4+.^15^ Consistently, we found that the Co valence states of the as prepared LSC/STO and LSC/LAO were dominated by Co^3+^[[qv: 12,14a,16]] based on the satellite peak position in Co 2p XPS spectra (Figure 4b). After vacuum annealing, the appearance of Co^2+^ satellite peaks can be clearly observed, providing the direct evidence of oxygen vacancy formation. It is notable that the LSC/STO with tensile strain showed more pronounced increase in Co^2+^ satellite peak intensity than LSC/LAO with compressive strain (Figure 4b), indicating more oxygen vacancies in LSC/STO. These results are in accord with the HRXRD results shown above. It is worth noting that the HRXRD and XPS are with very different probing depth. While XPS mainly probe the surface region of the films, the HRXRD provides average information from the whole films.

Confirming that oxygen vacancies were introduced into LSC thin films by vacuum annealing, the OER activities of the as‐prepared LSC films and annealed ones were compared by electrochemical measurement. The LSV tests were carried out continuously for 50 cycles to evaluate whether the changes in OER performance induced by oxygen defects are stable over long time operation. After vacuum annealing, both the LSC/LAO (with compressive strain) and the LSC/STO (with tensile strain) showed lower current density (left inset figure in Figure 4d) and larger Tafel slope (Figure S2, Supporting Information) than the as‐prepared samples, indicating a detrimental effect of the oxygen vacancies on the OER performance. The current density for the vacuum annealed LSC was found to increase over OER test, while the current density for the as‐prepared samples remained stable. The LSV results for the as‐prepared and vacuum annealed ones were very similar after the 50th cycle test, as shown in the right inset figure in Figure 4d. These results indicated that the negative impact of the oxygen vacancies on the OER performance of the LSC films can be recovered by refilling the oxygen back to the film during OER tests. The surface structure of all the films remained unchanged after vacuum annealing and OER test (Figure S5, Supporting Information), indicating all the impact of oxygen defect we observed above did not come from the surface area changes.

To understand the reason for the recovery of OER performance for vacuum annealed LSC (Figure 4d), the Co valance states of LSC films subjected to long time OER test were characterized ex situ by XPS. We observed the disappearance of Co^2+^ satellite peak in the Co 2p spectra for the vacuum annealed samples as shown in Figure 4c, indicating the refilling of surface oxygen vacancies during OER test (ACoO_3−δ_ + 2δOH^−^→ACoO_3_ + δH_2_O+2δe^−^). Consistently, HRXRD 2θ–ω scans results showed that the lattice of the vacuum annealed LSC films contracted after OER test (Figure S6, Supporting Information). Similar intercalations of oxygen into oxides in alkaline electrolyte were also observed previously in other oxygen deficient perovskite oxides.^17^ The oxygen diffusivity of LSC is estimated to be about 10^12^ cm^2^ s^−1^ at room temperature,^18^ suggesting that oxygen is sufficiently mobile in the material for recovery of OER activity.

All the HRXRD, XPS, and electrochemical measurements results consistently indicated that the presence of oxygen vacancies led to the degradation of OER activities for both the LSC/STO and the LSC/LAO. Such detrimental impact on the activity can be recovered by refilling oxygen back to the surface during OER test.

### 2.3 Coupling Effect Between Strain and Oxygen Defects and its Impact on the OER Activity of LSC

**Figure 5:**
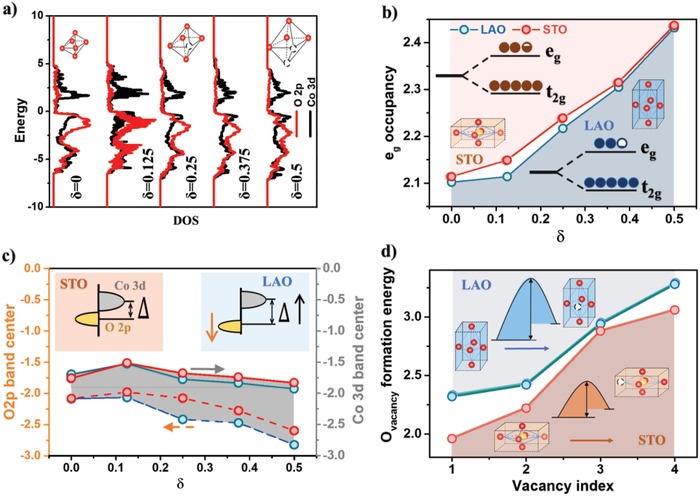
Comparison of the electronic structure and the oxygen vacancy formation energy for LSC/STO and LSC/LAO obtained in DFT calculations: a) Projected DOS of the O 2p and Co 3d states for LSC/LAO with different oxygen nonstoichiometry δ; b) the *e_g_* occupancy for the LSC with different δ value. The inset figure shows the slightly higher *e_g_* occupancy value for the LSC/STO; c) the O 2p and Co 3d band center for the LSC with different δ value. The inset figures show the larger energy gap between O 2p and Co 3d band center for the LSC/LAO; d) oxygen vacancy formation energy for the LSC with different number of oxygen vacancies in the unit cell. The inset figures show the smaller oxygen vacancy formation energy for the LSC/STO. The lines between data points shown in (b) and (c) are for guiding the eye.

To better understand the reason why LSC with compressive strain showed better OER activities and oxygen deficiency in LSC lead to worse OER performance, we carried out first‐principles calculations based on the density‐functional theory (DFT) to probe the electronic structures of LSC with various strain states and oxygen nonstoichiometry δ, and discussed how the difference in electronic structure impact the OER performance. **Figure** **5**a shows the representative calculation results of the density of states (DOS) for the LSC/LAO with different δ values. The results for LSC/STO are shown in Figure S5 in the Supporting Information. Two widely used electronic structure descriptors for OER activity were investigated systematically in this work: the occupancy of *e_g_* states (*e_g_* occupancy)[[qv: 7b,8b,19]] and the energy gap between O 2p and Co 3d band center (Δ)^20^ (Figure 5). It has been reported previously that the OER activity of perovskite oxides critically depend on its *e_g_* occupancy, with the optimal *e_g_* occupancy close to unity.[[qv: 7b,8b,19]] On the other hand, oxide catalysts with smaller Δ values were found to show higher electronic conductivity[[qv: 20a]] and more facile charge transfer process during OER reaction.[[qv: 20b]] Furthermore, previous work suggested that the OER on LSC can involve the exchange of lattice oxygen species,^18^, ^21^ and the increase in the covalency of metal–oxygen bonds, which can be quantified by a smaller energy gap between O 2p and Co 3d Δ or a higher O 2p‐band center position relative to the Fermi level, can promote the participation of lattice‐oxygen in OER and lead to higher OER activity of LSC.

We firstly compared the impacts of strain on the *e_g_* occupancy and Δ value of LSC for a δ value. As shown in Figure 5b, the *e_g_* occupancy for LSC/STO with tensile strain and LSC/LAO with compressive strain are quite close, with the *e_g_* occupancy for LSC/LAO to be slightly closer to the optimal value of unity. This result indicated that the LSC/LAO may show slightly more optimized bond strength to the reaction intermediates than the LSC/STO with the same δ, but such difference is not very significant. The Δ value, on the other hand, was found to be noticeably larger for the LSC/LAO than the LSC/STO with the same δ, suggesting lower electronic conductivity,[[qv: 20a]] harder charge transfer process,[[qv: 20b]] and less covalency of metal–oxygen bonds.^18^, ^21^ On the basis of the calculation results for *e_g_* occupancy and Δ value above, one should expect similar or even worse OER activity for the LSC/LAO than the LSC/STO for a given δ value, which is in contrast with the considerably better OER performance for LSC/LAO than LSC/STO we observed experimentally.

For both LSC films with tensile and compressive strain, our DFT results showed that the increase of δ lead to more electrons filled into the *e_g_* states (Figure 5b) and the increase of Δ value (Figure 5c). Such changes in electronic structure are likely to be the reason for the degraded OER activities for vacuum annealed LSC we observed in the electrochemical test.

As mentioned in the introduction section, lattice strain and oxygen defect chemistry are reported to be strongly coupled with each other.^9^ To understand such coupling effect for LSC, DFT calculations were carried out to determine the oxygen vacancy formation energy for the LSC/STO and the LSC/LAO using the strain value extracted from the HRXRD experiments. As shown in Figure 5d, the oxygen vacancy formation energy for the LSC/STO with tensile strain is considerably smaller than that for the LSC/LAO with compressive strain. These results are consistent with the higher O 2p band center for LSO/STO shown in Figure 5c, suggesting a smaller energy penalty for electrons to excite from O 2p band to either Co 3d band or near the Fermi level during oxygen vacancy formation.^22^ As a result of the smaller oxygen vacancy formation energy, the LSC/STO is likely to be equilibrium with more oxygen vacancies in the lattice than LSC/LAO under similar environments, leading to the larger δ value for LSC/STO as we observed in the HRXRD results. This conclusion is also supported by our XPS results which showed that LSC/STO exhibited more Co^2+^ than LSC/LAO after annealing, and is consistent with previous reports of Cai et al.[[qv: 9d]] The lower δ value in LSC/LAO than that in LSC/STO were likely to result in a considerably smaller *e_g_* states occupancy and a similar or even smaller Δ for LSC/LAO than LSC/STO. Both factors can eventually lead to the better OER activity of the LSC/LAO.

In this work, we found that the creation of excessive oxygen vacancies in LSC leads to lower OER activity, attributed to more deviation of *e_g_* filling from unity and larger energy gap between O 2p and Co 3d band. Similar detrimental effects of oxygen vacancies on the OER and ORR activity of La_0.6_Ca_0.4_CoO_3−δ_ were reported by Wu at el.[[qv: 8d]] For CaMn_0.75_Nb_0.25_O_3−δ_, on the other hand, the presence of oxygen vacancies was reported to enhance the OER and ORR activity, attributed to optimized *e_g_* filling, stronger OH^−^ adsorption, and improved electrical conductivity.[[qv: 8c]] Xu et al. reported significantly improved OER activity of Co_3_O_4_ with abundant oxygen vacancies created by plasma treatment, and believed the enhanced performance was due to the improved electronic conductivity and higher active defects density.[[qv: 8a]] All these results demonstrate that the effects of oxygen defects on the electrocatalytic activity of metal oxides depend critically on the material systems. It is critical to further investigate the role of oxygen defects for the rational design of high performance catalysts.

It is worth noting that we observed a decrease of surface Sr contents for both the LSC/STO and the LSC/LAO after OER test (Figure S8, Supporting Information), which is likely to be the reason for the activation process during the first few test cycles as shown in Figure S9 in the Supporting Information.^23^ Although previous works showed that surface chemistry can strongly impact the surface reaction kinetics of perovskite oxides,^24^ we did not observe noticeable difference in the surface composition between the LSC/STO and the LSC/LAO films. Therefore, we believe that the surface composition is not the major factor that leads to the different OER activity between the LSC/STO and the LSC/LAO. Furthermore, to understand the correlation among lattice strain, oxygen defects, and OER activity for LSC thin film, we calculated the *e_g_* occupancy (Figure 5b) and the Δ value (Figure 5c) as the function of strain and oxygen nonstoichiometry. There are other electronic structure descriptors for OER activity reported in literature.^25^ To survey all the electronic structure descriptors is beyond the scope of this manuscript. Our results, however, indeed have demonstrated the critical impact of strain–defect coupling effects on the electrocatalytic.

## 3 Conclusion

In this work, we investigated the effect of lattice strain and oxygen defect chemistry on the electrocatalytic activity of perovskite cobaltite oxide thin films using carefully designed experimental measurements and first‐principles calculations. We used model systems consisting of LSC thin films grown by PLD on STO and LAO single crystal substrates, with in‐plane tensile and compressive strain, respectively. Electrochemical tests showed that both the lattice strain and the oxygen defects play a critical role in determining the OER performance of the LSC thin films. The LSC/LAO with in‐plane compressive strain displayed considerably higher OER activities than the LSC/STO with in‐plane tensile strain. Both the LSC/STO and the LSC/LAO exhibited lower OER activities as more oxygen vacancies are introduced through vacuum annealing. Such degradation in OER performance can be recovered by refilling oxygen back to the films during OER test. Both experiments and DFT calculations suggested that the LSC/STO has larger oxygen deficiency than the LSC/LAO sample, attributed to its smaller oxygen vacancy formation energy. DFT calculation showed that such strain‐induced excessive oxygen vacancy in LSC/STO increased the *e_g_* state occupancy and enlarges the energy gap between O 2p and Co 3d band, resulting in lower OER activity. Engineering lattice strain and tuning defect chemistry can be used as effective approaches to achieve high‐performance catalysts. Our results demonstrated the critical role of strain–defect coupling effect in determining the electrocatalytic activity of perovskite oxides. Such understanding can be applied to other metal oxides and can be helpful for guiding the rational design of new catalysts for high‐performance energy devices.

## 4 Experimental Section

*Preparation of LSC Thin Film Electrodes*: The LSC model thin films with different strain states were deposited on LAO and STO single crystal substrates by PLD, using a KrF excimer laser with a wavelength of 248 nm and a pulse frequency of 10 Hz. The laser energy was set to 300 mJ per pulse and a target substrate distance of 10 cm was used. The deposition was carried out at 600 °C in 20 Pa oxygen gas environments. The samples were cooled down to room temperature in 2 × 10^2^ Pa oxygen with a cooling rate of 5 °C min^−1^. The thickness of the thin film was controlled by the number of laser pulse used in the deposition. The procedure to make PLD targets can be found in Section S2 in the Supporting Information.

*Electrochemical Characterization*: Before the deposition of LSC thin film, a gold pattern was sputtered onto the substrate, which was used as the current collector in the electrochemical measurement. (Figure 1a,b) The OER activities of the LSC thin films were evaluated using a three‐electrode electrochemical cell (Figure 1b), with the LSC thin film as the working electrode (WE), Pt as the counter electrode (CE), and Ag/AgCl as the reference electrode (RE). The electrochemical characterization was controlled using a Chi 660 Electrochemical workstation. A scan rate of 10 mV s^−1^ was used for all linear sweet voltammetry experiments. The 0.1 m KOH electrolyte was prepared using deionized water (>18 mΩ•cm) and KOH pellets. Oxygen bubbling and rapid stirring ensured O_2_/OH^−^ equilibrium at 1.23 V versus RHE.

*Material Characterization*: The crystal structure and the strain states of the LSC films were characterized by HRXRD 2θ–ω scans and RSM. The thickness of the LSC thin film was quantified by X‐ray reflectivity (XRR) measurements (Figure S10, Supporting Information). All these measurements were performed on Bruker D8 Discover diffractometer equipped with 2‐bounce Ge (220) channel‐cut monochromator using Cu Ka1 radiation. The surface morphology of the LSC thin films were characterized by SU8010 high resolution field emission scanning electron microscope (SEM) (Hitachi). The surface roughness was quantified by AFM in a tapping mode with MFP‐3D‐SA (Asylum Research). The surface composition and Co valence state were probed by XPS (US Thermo Fischer, ESCALAB 250Xi) with a Al kα source (hv = 1486.6 eV).

*Calculation*: First‐principles calculations were performed using Vienna ab‐initio Simulation Package (VASP).^26^ GGA‐PBE^27^ type exchange‐correlation functional was used, together with a 4 × 4 × 4 Monkhorst‐Pack^28^ k‐points and 600 eV energy cutoff. GGA +*U* with *U* = 4.0 eV for Co^29^ was used to account for Co's 3d electrons. LSC pseudo‐cubic cell contains 40 atoms, with 2 Sr replacing on La‐site. Five oxygen nonstoichiometry samples were considered, by creating 0–4 vacancies in the lattice. Lattice parameters of pseudo‐cubic, compressed and stretched LSC were adopted from experimental measurement. A spin‐state constrained scheme was used to screen over various spin states and locate the ground state. A preconditioned conjugate gradient method was used to update orbitals of all bands simultaneously.^30^

## Conflict of Interest

The authors declare no conflict of interest.

## Supporting information

Supplementary

## References

[1] D. Chen , C. Chen , Z. M. Baiyee , Z. Shao , F. Ciucci , Chem. Rev. 2015, 115, 9869. 26367275 10.1021/acs.chemrev.5b00073

[2] Y. Lee , J. Suntivich , K. J. May , E. E. Perry , Y. Shao‐Horn , J. Phys. Chem. Lett. 2012, 3, 399. 26285858 10.1021/jz2016507

[3] H. G. Sanchez Casalongue , M. L. Ng , S. Kaya , D. Friebel , H. Ogasawara , A. Nilsson , Angew. Chem., Int. Ed. 2014, 53, 7169. 10.1002/anie.201402311 24889896

[4] Y. Gorlin , T. F. Jaramillo , J. Am. Chem. Soc. 2010, 132, 13612. 20839797 10.1021/ja104587v

[5] X. Lu , J. Deng , W. Si , X. Sun , X. Liu , B. Liu , L. Liu , S. Oswald , S. Baunack , H. J. Grafe , C. Yan , O. G. Schmidt , Adv. Sci. 2015, 2, 1500113. 10.1002/advs.201500113 PMC5115390 27980974

[6] B. S. Yeo , S. L. Klaus , P. N. Ross , R. A. Mathies , A. T. Bell , ChemPhysChem 2010, 11, 1854. 20473978 10.1002/cphc.201000294

[7] a) K. A. Stoerzinger , W. S. Choi , H. Jeen , H. N. Lee , Y. Shao‐Horn , J. Phys. Chem. Lett. 2015, 6, 487; 26261968 10.1021/jz502692a

[8] a) L. Xu , Q. Jiang , Z. Xiao , X. Li , J. Huo , S. Wang , L. Dai , Angew. Chem., Int. Ed. 2016, 55, 5277; 10.1002/anie.201600687 26990905

[9] a) A. Herklotz , D. Lee , E. J. Guo , T. L. Meyer , J. R. Petrie , H. N. Lee , J. Phys.: Condens. Matter 2017, 29, 493001; 29130456 10.1088/1361-648X/aa949b

[10] X. Chen , T. Grande , Chem. Mater. 2013, 25, 927.

[11] E. Navickas , Y. Chen , Q. Lu , W. Wallisch , T. M. Huber , J. Bernardi , M. Stoger‐Pollach , G. Friedbacher , H. Hutter , B. Yildiz , J. Fleig , ACS Nano 2017, 11, 11475. 28981249 10.1021/acsnano.7b06228 PMC5707630

[12] Y. Chen , D. D. Fong , F. W. Herbert , J. Rault , J. P. Rueff , N. Tsvetkov , B. Yildiz , Chem. Mater. 2018, 30, 3359.

[13] a) J. G. Swallow , W. H. Woodford , Y. Chen , Q. Lu , J. J. Kim , D. Chen , Y. M. Chiang , W. C. Carter , B. Yildiz , H. L. Tuller , K. J. Van Vliet , J. Electroceram. 2014, 32, 3;

[14] a) N. Tsvetkov , Y. Chen , B. Yildiz , J. Mater. Chem. A 2014, 2, 14690;

[15] J. Mizusaki , Y. Mima , S. Yamauchi , K. Fueki , H. Tagawa , J. Solid State Chem. 1989, 80, 102.

[16] Y. Chen , Z. Cai , Y. Kuru , W. Ma , H. L. Tuller , B. Yildiz , Adv. Energy Mater. 2013, 3, 1221.

[17] a) J. T. Mefford , W. G. Hardin , S. Dai , K. P. Johnston , K. J. Stevenson , Nat. Mater. 2014, 13, 726; 24880729 10.1038/nmat4000

[18] J. T. Mefford , X. Rong , A. M. Abakumov , W. G. Hardin , S. Dai , A. M. Kolpak , K. P. Johnston , K. J. Stevenson , Nat. Commun. 2016, 7, 11053. 27006166 10.1038/ncomms11053 PMC4814573

[19] a) J. Suntivich , K. J. May , H. A. Gasteiger , J. B. Goodenough , Y. Shao‐Horn , Science 2011, 334; 22033519 10.1126/science.1212858

[20] a) X. Cheng , E. Fabbri , M. Nachtegaal , I. E. Castelli , M. El Kazzi , R. Haumont , N. Marzari , T. J. Schmidt , Chem. Mater. 2015, 27, 7662;

[21] A. Grimaud , O. Diaz‐Morales , B. Han , W. T. Hong , Y. L. Lee , L. Giordano , K. A. Stoerzinger , M. T. M. Koper , Y. Shao‐Horn , Nat. Chem. 2017, 9, 457. 28430191 10.1038/nchem.2695

[22] Y. L. Lee , J. Kleis , J. Rossmeisl , Y. Shao‐Horn , D. Morgan , Energy Environ. Sci. 2011, 4, 3966.

[23] L. C. Seitz , C. F. Dickens , K. Nishio , Y. Hikita , J. Montoya , A. Doyle , C. Kirk , A. Vojvodic , H. Y. Hwang , J. K. Norskov , T. F. Jaramillo , Science 2016, 353, 1011. 27701108 10.1126/science.aaf5050

[24] a) Y. Chen , W. Jung , Z. Cai , J. J. Kim , H. L. Tuller , B. Yildiz , Energy Environ. Sci. 2012, 5, 7979;

[25] W. T. Hong , R. E. Welsch , Y. Shao‐Horn , J. Phys. Chem. C 2016, 120, 78.

[26] G. Kresse , J. Furthmüller , Comput. Mater. Sci. 1996, 6, 15.

[27] J. P. Perdew , K. Burke , M. Ernzerhof , Phys. Rev. Lett. 1996, 77, 3865. 10062328 10.1103/PhysRevLett.77.3865

[28] H. J. Monkhorst , J. D. Pack , Phys. Rev. B 1976, 13, 5188.

[29] A. M. Ritzmann , J. M. Dieterich , E. A. Carter , Phys. Chem. Chem. Phys. 2016, 18, 12260. 27079696 10.1039/c6cp01720g

[30] T. A. Arias , M. C. Payne , J. D. Joannopoulos , Phys. Rev. Lett. 1992, 69, 1077. 10047117 10.1103/PhysRevLett.69.1077

