# Supplementary material for: Uncovering the Effect of Lattice Strain and Oxygen Deficiency on Electrocatalytic Activity of Perovskite Cobaltite Thin Films
Source: Adv Sci (Weinh). 2019 Jan 30;6(6):1801898. doi: 10.1002/advs.201801898 (PMC6425498; doi:10.1002/advs.201801898)
Supplement: Supplementary file 1 — Supplementary [file ADVS-6-1801898-s001.pdf]

Copyright WILEY-VCH Verlag GmbH & Co. KGaA, 69469 Weinheim, Germany, 2019.

ADVANCED SCIENCE
Open Access

# Supporting Information

for *Adv. Sci.,* DOI: 10.1002/advs.201801898

# Uncovering the Effect of Lattice Strain and Oxygen Deficiency on Electrocatalytic Activity of Perovskite Cobaltite Thin Films

*Xi Liu, Lei Zhang, Yun Zheng, Zheng Guo, Yunmin Zhu, Huijun Chen, Fei Li, Peipei Liu, Bo Yu, Xinwei Wang, Jiang Liu, Yan Chen,* and Meilin Liu**

Supporting Information

# Uncovering the Effect of Lattice Strain and Oxygen Deficiency on Electro-catalytic Activity of Perovskite Cobaltite Thin Films

Xi Liu, Lei Zhang, Yun Zheng, Zheng Guo, Yunmin Zhu, Huijun Chen, Fei Li, Peipei Liu, Bo Yu, Xinwei Wang, Jiang Liu, Yan Chen*, Meilin Liu*

*(Y. C.) E-mail address: escheny@scut.edu.cn

*(M. L.) E-mail address: meilin.liu@mse.gatech.edu

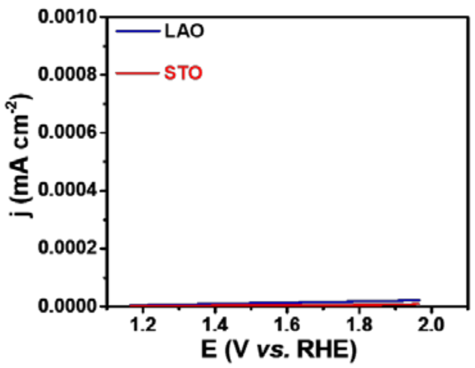


**Figure S1.** LSV curves for the OER on LAO and STO substrates. The substrate showed negligible contribution to the OER activity of the LSC thin film samples.

X. Liu, Y. Zhu, H. Chen, F. Li, Dr. P. Liu, Prof. J. Liu, Prof. Y. Chen
Guangzhou Key Laboratory for Surface Chemistry of Energy Materials, Guangdong Engineering and Technology and Research Center for Surface Chemistry of Energy Materials, State Key Laboratory of Pulp and Paper Engineering, School of Environment and Energy, South China University of Technology
Email: escheny@scut.edu.cn
L. Zhang, Prof. M. Liu
Materials Science and Engineering, Georgia Institute of Technology, Atlanta, USA
Email: meilin.liu@mse.gatech.edu
Y. Zheng, Prof. B. Yu
Institute of Nuclear and New Energy Technology, Tsinghua University, Beijing, China
Z. Guo, Prof. X. Wang
School of Advanced Materials, Shenzhen Graduate School, Peking University, Shenzhen, China

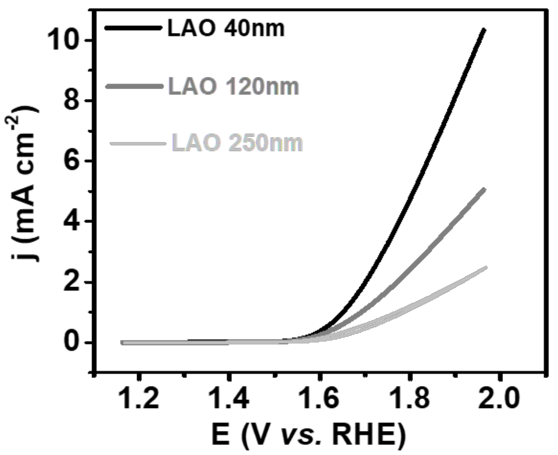


**Figure S2.** LSV curves for the OER on 40 nm, 120nm and 250 nm LSC films grown on LAO substrates

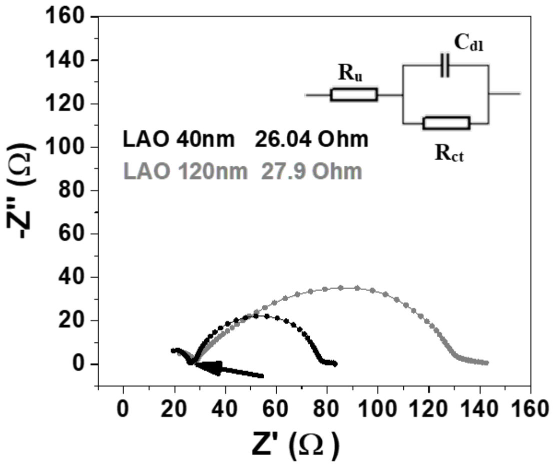


**Figure S3.** The resistances of liquid electrolytes obtained from electrochemical impedance spectroscopy for the 40nm and 120nm LSC/LAO films, which were found to be independence of the film thickness.

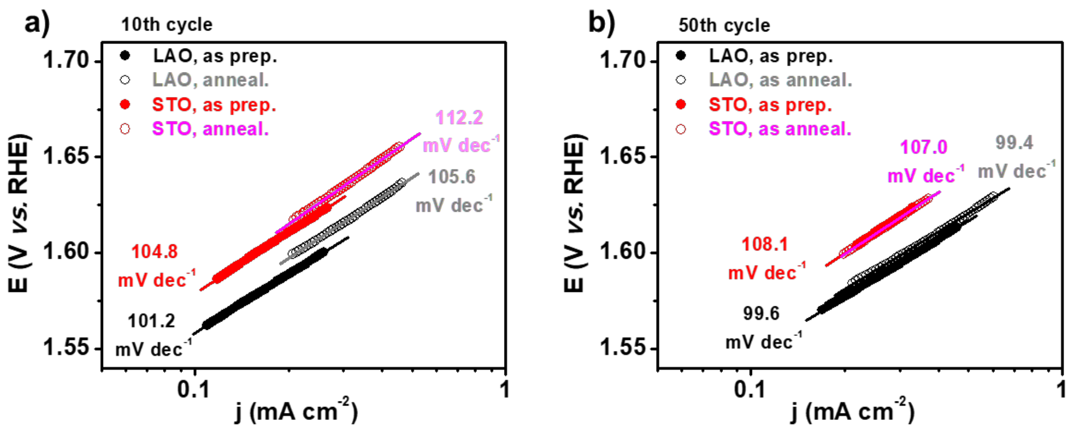


**Figure S4.** The Tafel slopes of the LSC thin films obtained from the 10th (a) and the 50th (b) LSV test for the OER activity.

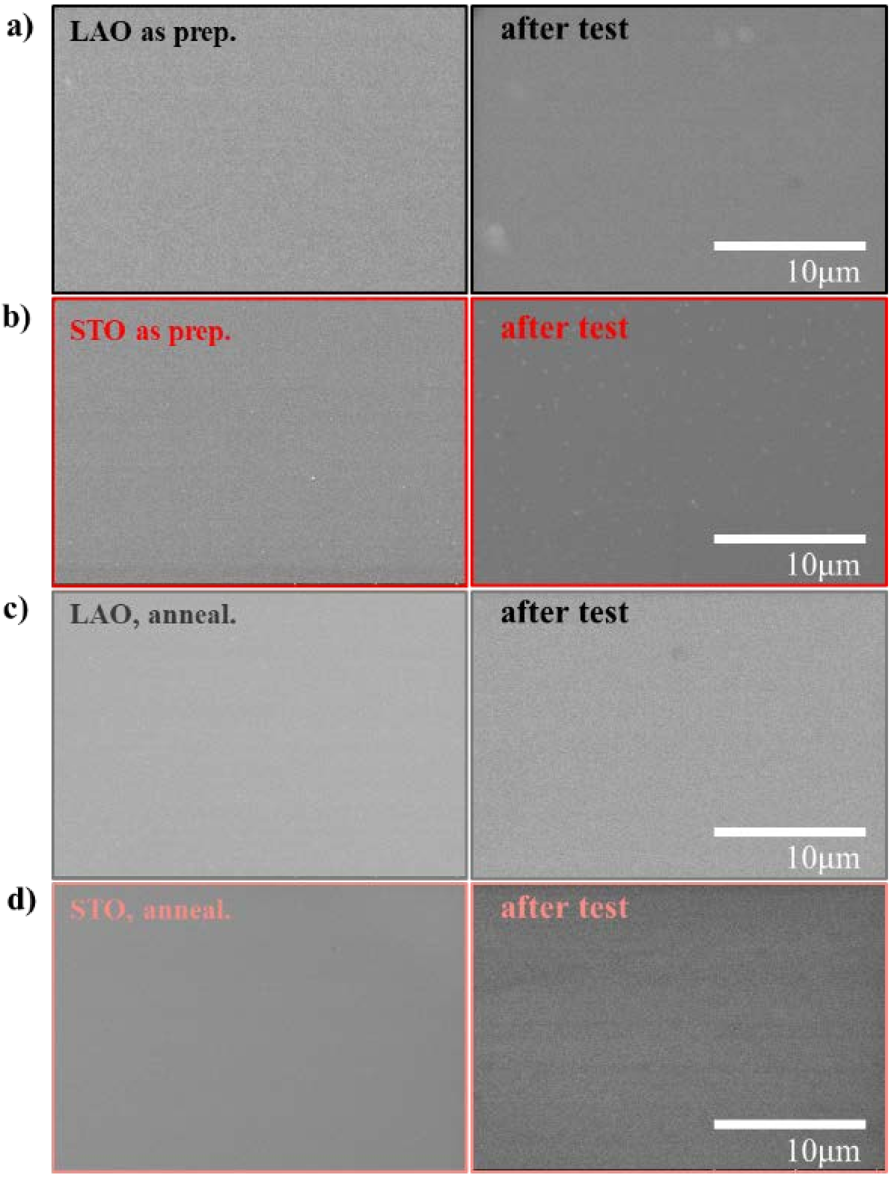


**Figure S5.** SEM images for the LSC films before and after OER tests: (a) as prepared LSC/LAO; (b) as prepared LSC/STO; (c) LSC/LAO after vacuum annealing; (d) LSC/STO after vacuum annealing. The surface morphology remained unchanged after being annealed in high vacuum condition at 300 $^{o}$C and after electrochemical test.

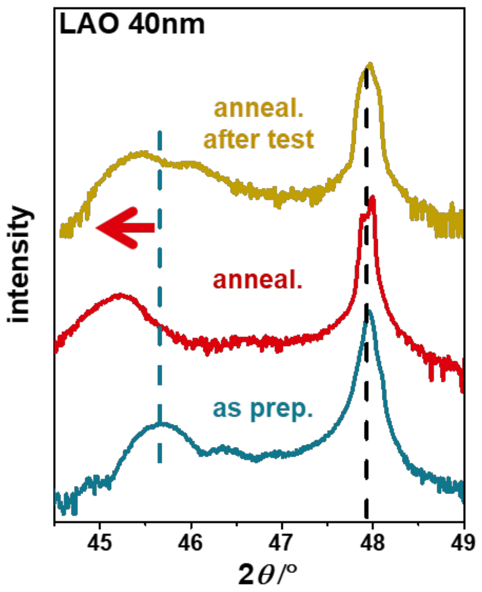


**Figure S6.** HRXRD 2θ-ω scans of the LSC/LAO in the as prepared state (green, as prep.), subjected to vacuum annealing (red, anneal.) and subjected to vacuum annealing and OER test (yellow, anneal. after test). The dash lines mark the peak position of the LAO substrate (black) and as prepared film (green).

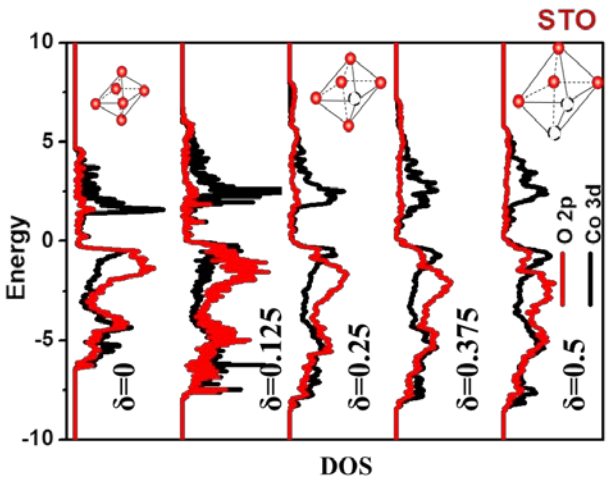


**Figure S7.** Projected density of states (DOS) for the O 2p and Co 3d states obtained by DFT calculations for the LSC/STO with different oxygen non-stoichiometry δ.

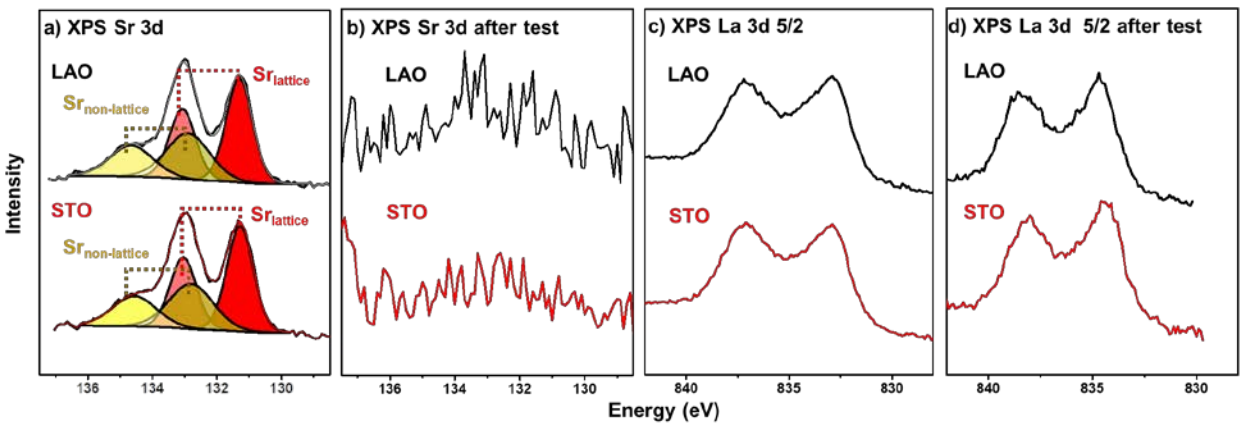


**Figure S8.** (a-b) Sr 3d spectra of the 40 nm LSC/LAO and LSC/STO before (a) and after OER test (b); the red and yellow doublets are attributed to bulk coordinated Sr in LSC lattice ($Sr_{lattice}$) and surface Sr species ($Sr_{non-lattic}$). The Sr contents decreased after OER test, indicating a dissolution of Sr species from the LSC films to the liquid electrolytes. (c-d) La 3d 5/2 spectra of the 40 nm LSC/LAO and LSC/STO before (c) and after OER test (d).

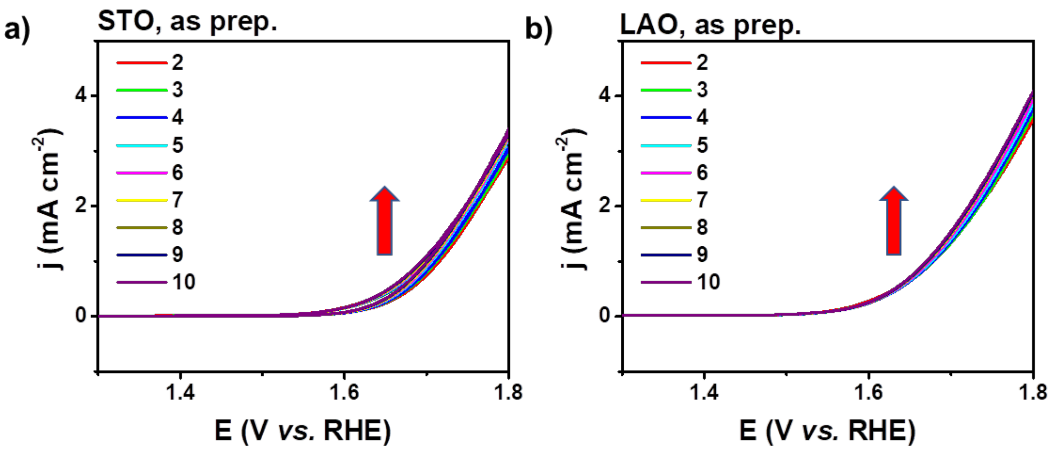


**Figure S9.** LSV curves for the 40nm LSC/STO (a) and LSC/LAO (b) obtained from the first few ten cycles. The current increased continuously suggesting an activation process occurs during the first few OER test cycles.

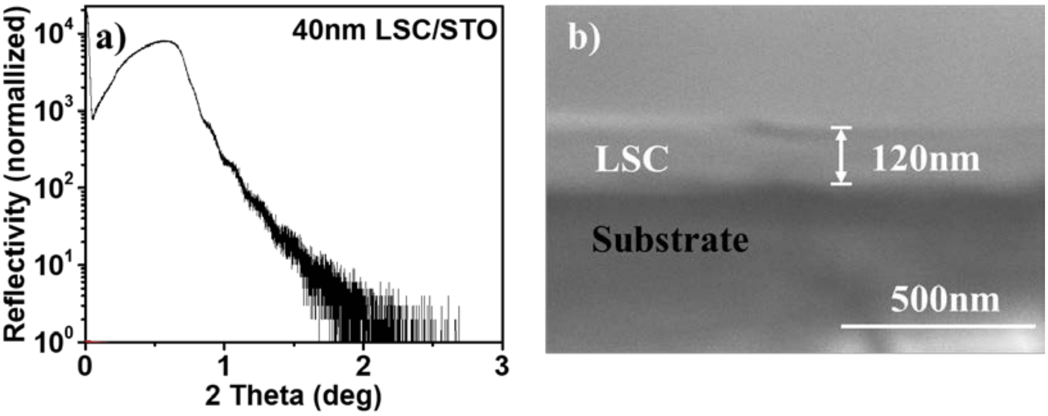


**Figure S10.** a) The XRR result of thin LSC film is about 40nm. b) The cross-section image of thick LSC film by SEM.

**S1: Oxygen non-stoichiometry of the LSC thin films estimated from HRXRD results**

The lattice volume of mixed electronic and ionic conducting oxides depends on both the temperature and oxidation state, which are normally described in terms of the oxygen-defect, i.e. oxygen vacancy and oxygen interstitial, concentration. Such dependencies can be quantified using thermal expansivity ($\beta_T$) and oxygen-vacancy chemical expansivity ($\beta_C$):

$$\beta_\mathrm{T} = \left(\frac{\partial \ln V}{\partial T}\right)_{x_\mathrm{v},p}, \quad \beta_\mathrm{C} = \left(\frac{\partial \ln V}{\partial x_\mathrm{v}}\right)_{T,p} \quad (1)$$

Where $V$ is the specific volume, T is temperature, P is total pressure and $x_v$ is the oxygen-vacancy mole fraction defined as $x_v$=δ/3, δ is the oxygen non-stoichiometry in $ABO_{3-\delta}$ (here we only consider the oxides with oxygen vacancy as the dominated defects).

When the thermal expansion is not considered for the case of constant temperature, one can estimated the changes in oxygen non-stoichiometry (Δ δ) based on the volume expansion (ΔV/V) and the oxygen-vacancy chemical expansivity ($\beta_C$) (equation (1)). Chen et al. systematically investigated the chemical expansion of polycrystalline $La_{1-x}Sr_xCoO_3$ with x= 0.2, 0.4, and 0.7.[1] They found the

chemical expansion in the LSC family of materials follows a “universal” correlation as the following:

$$\frac{1}{3}\beta_C(x_v) = 0.061 + 0.584 x_v \quad (2)$$

Applying the empirical correlation between $\beta_C$ and $x_v$ obtained by Chen et al. to our LSC thin films, we can get the following correlation between the change in the unit cell volume and the oxygen non-stoichiometry.

$$\left(\frac{\partial \ln V}{\partial \delta}\right)_{T,p} = 0.061 + \frac{0.584}{3}\delta \quad (3)$$

The relative changes in $\Delta\delta$ can be estimate based on the experimental $\Delta V/V$ value obtained from HRXRD (Table 1), with the assumption that the reference state (as prepared LSC/LAO) was with only very small amounts of oxygen vacancies.

**S2: Pulsed Laser Deposition Target synthesis**

LSC powder was synthesized using a solid-state method. Stoichiometric amounts of $La_2O_3$ (which was calcined at 1000 °C in air for 2 h before weighting to remove absorbed water), $SrCO_3$ and $Co_3O_4$ were dissolved in ethanol and mixed by ball milling for 8 h, and the mixtures were then calcined at 1000 °C in air for 4 h to form a perovskite LSC phases. To preparation of LSC target, the LSC powder was grinded with PVB-ethanol solution for 30 min, and then was pressed into a pellet under a pressure about 300 MPa . The pellet was calcined at 1100 °C in air for 12 h to form the PLD target.

[1] X. Y. Chen, J. S. Yu, S. B. Adler, *Chemistry of Materials* **2005**, 17, 4537.
